# A within-subject examination of grandparents’ physical activity and sedentary behavior levels in the presence or absence of grandchild care provision

**DOI:** 10.1186/s11556-024-00345-8

**Published:** 2024-05-07

**Authors:** Maxine Vanhove, Eva D’Hondt, Yanni Verhavert, Tom Deliens, Benedicte Deforche, Marie Vermote

**Affiliations:** 1 Department of Movement and Sport Sciences, Vrije Universiteit Brussel, Pleinlaan 2, Brussels, 1050 Belgium; 2 Department of Public Health and Primary Care, Ghent University, C. Heymanslaan 10, Ghent, 9000 Belgium; 3 Research Foundation – Flanders (FWO), Leuvenseweg 38, Brussel, 1000 Belgium

**Keywords:** Healthy Grandparenting Project, Healthy aging, Energy-expenditure related behavior, Intergenerational care, Older adults

## Abstract

**Background:**

This study aimed to examine within-subject differences in levels of physical activity (PA) and sedentary behavior (SB) among Flemish grandparents aged 50 years and older during a day of providing versus not providing grandchild care. Additionally, grandparents’ PA and SB levels of the specific caregiving moment within the included care day were also compared with those of the corresponding specific time frame on the matching non-care day.

**Methods:**

Data were obtained and pooled from three assessment time points of the Healthy Grandparenting Project. Objectively measured PA and SB levels were assessed through ActiGraphs wGT3x(+) worn during waking hours for seven consecutive days and expressed relative to the total wear time of the selected days or moments (i.e., percentage of time per day or per moment). Generalized linear mixed models were used to evaluate the within-subject differences in grandparents’ light intensity PA (LIPA), moderate-to-vigorous intensity PA (MVPA) and SB levels between a care and non-care day as well as between the care and non-care moment of those respective days.

**Results:**

A total of 92 grandparents (64.6 ± 4.8 years, 67.4% women) were included in the analyses. During the care day and care moment, grandparents showed higher relative levels of LIPA (∆=4.0% and ∆=7.9%, respectively) and lower relative levels of SB (∆=3.7% and ∆=6.7%, respectively) as compared to their respective non-care day and non-care moment (all *p* < 0.001). While there was no significant difference in relative MVPA levels between a day of providing versus not providing grandchild care (∆=0.3%, *p* = 0.500), the grandparents showed significantly lower relative levels of MVPA during the specific care moment against the non-care moment (∆=1.3%, *p* = 0.029).

**Conclusions:**

The higher percentage of time of LIPA and lower percentage of time spent on SB during a care day and care moment compared to a non-care day and non-care moment, highlight the positive impact of grandchild care provision on grandparents’ activity levels, potentially improving other health-related outcomes. Furthermore, grandparents seem to compensate for their lower MVPA levels during the actual care moment since no differences in MVPA levels were found at day level when compared to a day without grandchild care.

**Trial registration:**

clinicaltrials.gov, Identifier: NTC04307589. Registered March 2020.

## Background

Over the last decades, the share of aging adults in the general population has increased substantially [1]. A large part of this increase can be attributed to improved living conditions, such as better hygiene, sufficient food supply as well as improved medical and health care [2–4]. If the current demographic trend of our aging society continues, 30.3% of the European population is expected to be aged at least 65 years and 13.2% to be aged 80 years and older, by 2070 [5]. As a result, there has been a notable upsurge in research interest on healthy aging lately, being defined as “the process of developing and maintaining the functional ability that enables well-being in older age” [6].

Additional socio-demographic trends, such as more women in the labor force and a higher rate of divorces, have resulted in an increased demand for child care [7–9]. Furthermore, public services of formal child care (e.g., nurseries, independent daycare professionals and/or childminders) are becoming more and more scarce [7, 8, 10, 11]. Therefore, parents often have to find alternative care options, especially for their young(er) children [7]. Furthermore, family relationships across more than two generations are becoming increasingly important in today’s aging societies [12], with grandparents tending to play a significant role in providing care for their grandchildren [7, 13]. As grandparents make up a large part of the middle-aged and older adult population [14], and given the importance of healthy aging [6], considerable research attention has been paid to grandparents’ health in relation to their caregiving activities. Furthermore, grandchild care provision has been associated with better physical and mental health, improved cognitive functioning and overall well-being in grandparents [15–18].

As a specific health-promoting behavior, physical activity (PA) has been proven to contribute to the prevention of several non-communicable diseases (e.g., cardiovascular diseases, cancer, diabetes) [19]. Furthermore, adequate levels of PA seem to prevent falls in elderly and could retain and/or improve their cognitive and functional ability [19]. As such, being sufficiently physically active has been proven to increase the likelihood of healthy aging by 39%, and also to facilitate the maintenance of one’s general well-being later in life [20, 21]. Yet, most adults over the age of 50 years do not meet the weekly recommended guidelines of aerobic activity volume (i.e., engaging in moderate-intensity aerobic PA (MPA) for 150–300 min per week or 75–150 min of vigorous-intensity aerobic PA (VPA) per week) [19, 22, 23]. On top of being sufficiently physically active, it is also important for middle-aged and older adults to decrease and regularly interrupt their sedentary behavior (SB) [23]. Earlier research has shown that those adults who exhibit lower levels of SB were more likely to age in a healthy manner, given the association with less overweight/obesity as well as a decreased mortality risk resulting from a variety of diseases [24]. However, evidence also indicated that more than two-thirds of the aging adult population spend 60–80% of their waking hours while sitting or lying down, which corresponds to no less than 8.5 h per day [25]. Therefore, it is important to monitor and better understand variations in both PA and SB levels in view of healthy aging [26].

Given that providing care for one’s grandchildren may lead to the performance of new and/or additional (physical) tasks, such as lifting or carrying, feeding, bathing them as well as playing (on the floor) and/or going for a walk together, it could influence grandparents’ health indirectly through associated changes in PA and SB levels [27]. Yet, little is known about the relationship of providing care for grandchildren on grandparents’ energy-expenditure related behavior. Preliminary results of the Healthy Grandparenting Project (HGP), in which the impact of providing non-residential grandchild care on levels of PA and SB in people aged 50 years and older was examined, indicated that caregiving grandparents (i.e., grandparents who provide care for their grandchildren more than once a month) showed higher amounts of light intensity PA (LIPA) and lower amounts of SB on a weekly basis as compared to non-caregiving grandparents (i.e., grandparents who provide care for their grandchildren only once a month or less) and non-grandparents [28]. However, it remains unclear whether these between-subject differences in higher levels of LIPA and lower levels of SB among the caregiving grandparents were obtained during the actual provision of care for their grandchildren (as a direct result) or whether they were more active during their own free time when not being in charge of grandchild care (as an indirect result). In order to better understand the possible impact of providing grandchild care on both grandparents’ PA and SB levels, it is essential to investigate this in more detail.

Therefore, the aim of the present study was to address this gap and scrutinize within-subject differences in the levels of PA and SB among grandparents aged 50 years and older on a day of providing grandchild care versus a day without providing grandchild care (i.e., care day vs. non-care day) in a non-residential setting. Moreover, grandparents’ levels of PA and SB of the respective care moment during the included care day (i.e., care moment) were compared to the corresponding time frame on the matching non-care day (i.e., non-care moment).

## Methods

### Study design and participants

This comparative study was conducted using data from the HGP, which investigated the impact of providing non-residential grandchild care on PA and SB levels in people aged 50 years and older. Participants for this overarching HGP were recruited from June to August 2021 through various ways, including (social) media advertisement (e.g., Facebook) and via elderly movements in Flanders (e.g., Gezinsbond, OKRA 55+), for which a flyer was created and distributed. Additionally, local preschools and child day care centers were contacted to facilitate the recruitment of participants by distributing flyers to the children’s (grand)parents. In view of this larger HGP, individuals aged 50 years and older, with and without non-residential grandchildren, were recruited through convenience sampling. The HGP used a prospective cohort study design, conducting measurements at three assessment time points, each separated by a 6-month time interval. The current study used pooled data from each of these HGP assessment time points (i.e., T0, T1, and T2) situated between September 2021 and December 2022. This resulted in an increased number of observations, potentially amplifying the statistical power of the analyses. Only grandparents who provided written informed consent before the start of the study were considered for inclusion. Additionally, eligibility for the current study required these grandparents to provide care for one or more grandchild(ren), with at least one child aged between 0 and 5 years, during the data collection week of each assessment time point. Details of the sampling frames, methodology, assessments and the self-report questionnaire used in the overarching HGP have been reported elsewhere [29]. The HGP study protocol was approved by the Medical Ethics Committee of the local University Hospital (UZ Brussel, Brussels, Belgium; B.U.N. 1432020000017). The study was conducted according to the guidelines of the Declaration of Helsinki and its later amendments.

### Procedure and measurements

The majority of participating grandparents of the HGP indicated that in a typical week they provided care for their grandchildren once a week, encompassing non-residential and supplementary care. Therefore, this once a week frequency serves as the rationale for comparing one care day with one day without care in the present study [28]. Furthermore, at each assessment time point of the HGP, all measurements were performed by the principal investigator (i.e., MV, last author of this manuscript) during visits at the participants’ homes. During these home visits, the participating grandparents’ anthropometric characteristics (i.e., body height and body weight) were objectively determined [29]. Moreover, participants were instructed to wear an ActiGraph wGT3X(+) for seven consecutive days in order to obtain objective PA and SB data, starting from the day after the home visit. During each assessment week, the grandparents had to keep a diary in which they had to register the moments when they took off the accelerometer (e.g., during water-based activities). Furthermore, the grandparents were also asked to register all moments of grandchild care in the absence of the children’s parent(s) during each assessment week. An additional self-report questionnaire was used to obtain socio-demographic information of both the grandparents and their grandchild(ren). All accelerometer devices, completed diaries and questionnaires were received by postal return.

### Anthropometrics and Socio-demographic characteristics

Various anthropometrics of the grandparents were measured during the home visit, according to the guidelines of the International Society for the Advancement in Kinanthropometry [30]. Both body height (up to the nearest 0.1 cm) and body weight (up to the nearest 0.1 kg) were determined using a SECA 213 and TANITA MC-780MA S, respectively. From these results, participants’ body mass index (BMI, in kg/m²) was calculated, by dividing their body weight by their squared body height. Furthermore, a self-report questionnaire was used to obtain socio-demographic information about the grandparents and their grandchild(ren). Participants’ date of birth, sex and employment status were questioned, as well as the number of grandchildren within the family at the moment of the home visit and the corresponding birth date and biological sex of each of these grandchildren. The socio-economic status (SES) of the grandparents was estimated based on their highest educational attainment [31]. Participants who completed higher educational studies were classified as ‘higher SES’, while those with lower educational attainment were categorized as ‘lower SES’ [31].

### Physical activity (PA) and sedentary behavior (SB)

Participants’ PA and SB levels were objectively measured by means of a hip-worn ActiGraph wGT3X(+). This instrument already demonstrated excellent validity and reliability in terms of assessing PA and SB in middle-aged and older adults [32]. The accelerometer, which was attached to an elastic band, was worn at the height of the iliac crest on the right side of the body with the black button facing upwards. The grandparents were instructed to wear the accelerometer all day, during the seven consecutive days following each home visit, except when sleeping, performing water-based activities or activities where a disturbing effect could be experienced by wearing the device (e.g., yoga, karate). Furthermore, the participants were asked to keep a diary to register all activities performed during their waking hours when not wearing the accelerometer along with the duration and the self-experienced intensity of those activities (i.e., light, moderate or vigorous). In addition, they were asked to write down the time of getting up and going to sleep. By generating an activity count proportional to the measured acceleration in epochs of 30s, the ActiGraph accelerometer measured the frequency and duration of PA and SB as well as the intensity of PA.

### Data processing and terminology

As the purpose of this study was to compare grandparents’ levels of PA and SB on a day when grandchild care was provided (i.e., care day) versus a day without providing grandchild care (i.e., non-care day), it was decided to compare one care day with one matching non-care day within each subject per assessment time point. The selection of the respective care day and non-care day from each data collection week was based on the information obtained from the completed grandchild care diaries. These diaries provided us with information on the date of the grandchild care moment, the duration of grandchild care as well as the number of grandchildren simultaneously cared for, together with the sex and the age of those grandchildren. In view of the present study, a care day was defined as a weekday on which at least two consecutive hours of grandchild care were provided to one or more grandchild(ren), including at least one grandchild aged 5 years or younger, in the absence of the child(ren)’s parent(s). Days with less than two hours of consecutive grandchild care or weekend days (which usually involve fewer routine activities compared to weekdays, possibly impacting daily PA and SB levels) were not included. In addition, only days during which the accelerometer was worn uninterruptedly were included. In order to be consistent, the first care day of each assessment week (i.e., when the grandparent was wearing the accelerometer device) fulfilling all of the abovementioned grandchild care criteria was determined as the care day. A non-care day was defined as a weekday on which no care was provided at all to any grandchild and during which the accelerometer was also worn continuously. Additionally, a matching non-care day of which the accelerometer total wear time was closest to that of the selected care day was then included for within-subject comparative purposes.

For each selected care day, particular attention was given to the specific time frame during which grandchild care was actually provided as recorded by the grandparents in their diaries (i.e., care moment). To ensure consistency, this care moment was subsequently compared with the corresponding time frame on the included non-care day (i.e., non-care moment) for each of the grandparents.

After the selection of the care day and non-care day as well as the respective care moment and non-care moment within each participant per assessment time point, the levels of PA and SB of these specific days and moments were calculated using ActiLife software version 6.13.4 based on the collected raw accelerometer data. All PA related outcome measures (i.e., LIPA, MPA and VPA) were expressed relative to the total wear time of the day or the total wear time of the moment (i.e., percentage of time per day or percentage of time per moment, respectively). All values were calculated conform the protocol of Freedson et al. [33], with LIPA corresponding to 101–1951 counts per minute, MPA corresponding to 1952–5724 counts per minute, and VPA as > 5724 counts per minute. For the current study, participants’ levels of moderate-to-vigorous intensity PA (MVPA) were calculated by summing the values of MPA and VPA. Furthermore, SB was defined as < 101 counts per minute [33]. SB related outcome measures were also expressed relative to the total wear time (i.e., percentage of time per day or percentage of time per moment) during waking hours of the day or the moment under investigation, respectively.

### Statistical analysis

All data were analyzed using R (R Studio version 4.2.3). Multilevel mixed models were used to assess within-subject differences in LIPA, MVPA and SB between the care day and non-care day as well as between the care moment and non-care moment for each grandparent. Two-level models (i.e., repeated measures clustered within participants) were applied for each analysis. The distributions of the dependent variables (i.e., LIPA, MVPA and SB percentages) were first checked using histograms and QQ-plots. The values of LIPA and SB were found to be normally distributed for both days and moments, whereas MVPA values were non-normally distributed (i.e., positively skewed) for both days and moments. Therefore, generalized linear mixed models (GLMMs) with Beta variance and logit link functions were constructed for the LIPA and SB percentages and GLMMs with Gamma variance and log link functions were applied for the MVPA percentages using the lmer() and glmmTMB() function of the R *packages lme4* [34] and *glmmTMB* [35], respectively. In case of convergence problems or a singular fit, greater accuracy for evaluating the adaptive Gauss-Hermite approximation to the log-likelihood was allowed. To this end, the nAGQ-argument of the glmer function was set to two or more [34]. Note that this argument defaults to one, corresponding to the Laplace approximation [34]. In total, six separate models were constructed, including LIPA, MVPA or SB as an outcome variable compared between the care day versus non-care day as well as the care moment versus non-care moment within participants. Since pooled data from the HGP’s T0, T1, and T2 were used in the analyses, considering the potential influence of these different assessment time points on the within-subject difference in PA and SB levels between the care day and non-care day as well as the care moment and non-care moment, all models were controlled for this aspect. When there was an influence of the difference in assessment time points, the difference for the respective outcome variable according to the provision of care (per day or per moment) was separately analyzed within each assessment time point. In all other cases, the differences were examined regardless of assessment time point. Data visualization was performed by using the ggplot2-package [36] and the sjPlot-package [37] based on the predicted values of the outcome variable. The models used for our statistical analyses were not corrected for any of the grandparents’ characteristics (e.g., their age or sex), as it turned out not to be necessary to correct analyses for participants’ characteristics as these will not differ within a person in a relative short measurement period of one week (per assessment time point). For all analyses, *p-*values ≤ 0.05 were considered statistically significant.

## Results

After applying the in- and exclusion criteria, data from 92 grandparents who met the predefined criteria were retained for the current study. Among these participants, 36 grandparents participated at only one assessment time point (i.e., 36 within-subject comparisons), 38 grandparents participated at two assessment time points (i.e., 76 within-subject comparisons), and 18 grandparents participated at all three assessment time points (i.e., 54 within-subject comparisons). This resulted in a total of 166 within-subject comparisons (i.e., 166 care vs. non-care day comparisons as well as 166 care vs. non-care moment comparisons) of grandparents’ PA and SB levels. Appendix I provides an overview on how the individual data entries were distributed over the different assessment time points (see Table A). The participant selection process for each assessment time point is described in more detail in Appendix II (see Table B).

Our total study sample of 92 grandparents, providing care for their grandchild(ren), had an overall mean age of 64.6 ± 4.7 years and consisted for 67.4% out of women. Furthermore, 57.6% of the total study sample had a normal BMI (i.e., 18.5–24.9 kg/m^2^), 68.5% had a higher SES and 73.9% of the participating grandparents were already retired. On average, grandparents provided care for one or two grandchildren simultaneously, who had a mean age of 3.4 ± 2.0 years. More detailed study sample characteristics are presented in Tables 1 and 2.

The average duration of a day on which grandchild care was provided, as indicated by the total ActiGraph wear time, was 915.6 ± 77.9 min (i.e., 15.3 ± 1.3 h). On a day on which no grandchild care was provided, this total duration averaged 910.0 ± 63.3 min (i.e., 15.2 ± 1.1 h). Furthermore, the average duration of an equally time-framed care and non-care moment was 342.3 ± 187.0 min (i.e., 5.7 ± 3.1 h) (Table 2). Appendix III presents the average duration of time spent by our included participants in the different PA intensity levels and SB during an average care vs. non-care day (see Table C) as well as during an average care vs. non-care moment (see Table D) over the 166 observations. Moreover, Appendix IV shows boxplots to visually depict the variations in care day and non-care day total wear times and the variations in care moment and non-care moment durations, allowing for a clearer understanding of the data distribution, also including the minimum and maximum values.

**Table 1:** Characteristics of the total study sample of individual participants (*N* = 92)

Characteristics	Percentage or mean ± SD	
Sex		
Female (%)	67.4	
Age (years)	64.6 ± 4.7	
BMI (kg/m^2^)	24.9 ± 3.5	
Underweight (%)	0.0	
Normal weight (%)	57.6	
Overweight (%)	31.5	
Obese (%)	10.9	
Employment status		
Retired (%)	73.9	
Socioeconomic status (SES)		
Higher (%)	68.5	

N: number of participants; BMI: body mass index; SES: socio-economic status; SD: standard deviation. The above results were calculated using the baseline data of the participants at their initial assessment time point

**Table 2:** Grandchild characteristics and durations of the within-subject comparisons based on all individual data entries across the three assessment time points (*N* = 166)

Characteristics	Percentage or mean ± SD
Average number of GC^a^ per GP	1.6 ± 0.8
Mean age of GC^a^ (years)	3.4 ± 2.0
Combination of sex of GC^a^	
Only boys (%)	44.0
Only girls (%)	30.1
Combination (%)	25.9
Duration of care day (min.day^− 1^)	915.6 ± 77.9
Duration of non-care day (min.day^− 1^)	910.0 ± 63.3
Duration of care moment (min.day^− 1^)	342.3 ± 187.0
Duration of non-care moment (min.day^− 1^)	342.3 ± 187.0

N: number of observations / within-subject comparisons; GP: grandparent; GC: grandchild; SD: standard deviation; ^a^: grandchild(ren) specifically being cared for on the care days selected for analyses

### Care day versus non-care day

Table 3 presents the crude means and standard deviations of relative LIPA, MVPA and SB levels (expressed as a percentage of the total wear time) during the care day and the non-care day. For the comparison of LIPA levels, significantly higher levels were found during the care day compared to the matching non-care day at T0 (∆5.8%; *p* < 0.001) and at T1 (∆4.5%; *p* < 0.001) (see Table 4). Regardless of time point, the comparison of MVPA levels showed no significant difference between the care day and the non-care day (∆0.3%; *p* = 0.500) (see Table 4). Additionally, when comparing grandparents’ SB levels between the care day and the non-care day, grandparents demonstrated lower SB levels during the care day compared to the non-care day both at T0 and T1 (∆6.0%, *p* < 0.001; ∆4.3%, *p* < 0.001; respectively) (see Table 4).

**Table 3:** Crude means of PA and SB levels during a care day and a non-care day

Outcome variables	Mean ± SD
**Light intensity physical activity (LIPA) (% of time/day)**	
Care day	33.5 ± 8.6
Non-care day	29.5 ± 9.4
**Moderate-to-vigorous intensity physical activity (MVPA) (% of time/day)**	
Care day	3.7 ± 3.3
Non-care day	4.0 ± 4.1
**Sedentary behavior (SB) (% of time/day)**	
Care day	62.8 ± 9.2
Non-care day	66.5 ± 10.0

SD: standard deviation; LIPA: light intensity physical activity; MVPA: moderate-to-vigorous intensity physical activity; SB: sedentary behavior. The symbol “%” is used to indicate a percentage. In this case, these percentages indicate the proportion of total time spent on physical activity (PA) and sedentary behavior (SB) relative to the total wear time (i.e., care day or non-care day). It indicates the proportion of time spent on PA and SB relative to 100 (i.e., corresponding with the total accelerometer wear time during waking hours) and can help provide a general understanding of how much time was spent on these different activity levels

**Table 4:** Differences in PA and SB levels during a care day vs. a non-care day

	Estimates (95% CI)	*p*-value
**Light intensity physical activity (LIPA)** ^a^		
Non-care day (Ref. = Care day)		
T0	0.75 (0.67; 0.84)	< 0.001*
T1	0.81 (0.73; 0.90)	< 0.001*
T2	0.93 (0.82; 1.05)	0.240
**Moderate-to-vigorous intensity physical activity (MVPA)** ^b^		
Non-care day (Ref. = Care day)	1.08 (0.86; 1.35)	0.500
**Sedentary behavior (SB)** ^a^		
Non-care day (Ref. = Care day)		
T0	1.31 (1.17; 1.48)	< 0.001*
T1	1.21 (1.09; 1.35)	< 0.001*
T2	1.04 (0.92; 1.17)	0.538

^a^ Generalized linear mixed models with Beta variance and logarithmic link functions; ^b^ Generalized linear mixed models with Gamma variance and logarithmic link functions; Ref.: reference category; Values represent exponentiated regression coefficients and confidence intervals; *: significantly different (*p* ≤ 0.05) from the care day

### Care moment versus non-care moment

Table 5 presents the crude means and standard deviations of relative LIPA, MVPA and SB levels (expressed as a percentage of the total moment time) during the care moment and the corresponding non-care moment. For the comparison of LIPA levels according to the provision of grandchild care at the specific time frame within the day, significantly higher levels were found during the care moment compared to the non-care moment (∆=7.9%; *p* < 0.001) (see Table 6). Concerning the comparison of MVPA levels, grandparents exhibited significantly more percentage of their time in MVPA during the non-care moment compared to the care moment (∆=1.3%; *p* = 0.029) (see Table 6). Additionally, when comparing grandparents’ SB levels between the care moment and the non-care moment, significantly lower levels were found during the care moment compared to the corresponding non-care moment (Δ = 6.7%; *p* < 0.001) (see Table 6).

**Table 5:** Crude means of PA and SB levels during a care moment and a non-care moment

Outcome variables	Mean ± SD
**Light intensity physical activity (LIPA) (% of time/moment)**	
Care moment	40.0 ± 11.9
Non-care moment	32.1 ± 13.8
**Moderate-to-vigorous intensity physical activity (MVPA) (% of time/moment)**	
Care moment	4.2 ± 6.9
Non-care moment	5.5 ± 8.1
**Sedentary behavior (SB) (% of time/moment)**	
Care moment	55.7 ± 12.9
Non-care moment	62.4 ± 15.6

SD: standard deviation; LIPA: light intensity physical activity; MVPA: moderate-to-vigorous intensity physical activity; SB: sedentary behavior. The symbol “%” is used to indicate a percentage. In this case, these percentages indicate the proportion of total time spent on physical activity (PA) and sedentary behavior (SB) relative to the total moment time (i.e., care moment or non-care moment). It indicates the proportion of time spent on PA and SB relative to 100 (i.e., corresponding with the total accelerometer wear time during the specific moment) and can help provide a general understanding of how much time was spent on these different activity levels

**Table 6:** Differences in PA and SB levels during a care moment vs. a non-care moment

	Estimates (95% CI)	*p*-value
**Light intensity physical activity (LIPA)** ^a^		
Non-care moment (Ref. = Care moment)	0.75 (0.67; 0.85)	< 0.001*
**Moderate-to-vigorous intensity physical activity (MVPA)** ^b^		
Non-care moment (Ref. = Care moment)	1.31 (1.03; 1.57)	0.029*
**Sedentary behavior (SB)** ^a^		
Non-care moment (Ref. = Care moment)	1.33 (1.19; 1.50)	< 0.001*

^a^ Generalized linear mixed models with Beta variance and logarithmic link functions; ^b^ Generalized linear mixed models with Gamma variance and logarithmic link functions; Ref.: reference category; Values represent exponentiated regression coefficients and confidence intervals; *: significantly different (*p* ≤ 0.05) from the care moment

## Discussion

As part of the larger HGP, the present study examined within-subject differences in PA and SB levels among Flemish grandparents aged 50 years and older between a day of providing grandchild care (i.e., care day) versus not providing grandchild care (i.e., non-care day) as well as during the actual care moment versus the corresponding non-care moment (within the selected care and matching non-care day).

When comparing grandparents’ relative LIPA and SB levels during the care moment versus the non-care moment, grandparents turned out to obtain significantly higher levels of LIPA and lower levels of SB when providing grandchild care. Moreover, the same significant within-subject differences in terms of higher LIPA and lower SB levels on the care day against the non-care day were observed at T0 and T1, although these differences were less pronounced than when comparing the respective moments during these days. These observations are complementary with the preliminary findings of an earlier case-control study conducted within the HGP, indicating that caregiving grandparents (i.e., grandparents who provide care for their grandchildren more than once a month) showed higher amounts of LIPA and lower amounts of SB on a weekly basis compared to non-caregiving grandparents (i.e., grandparents who provide care for their grandchildren only once a month or less) and non-grandparents [28]. Therefore, the results of the current study can indicate that these elevated LIPA levels and reduced SB levels observed in caregiving grandparents are likely to be a direct result of providing care for their grandchildren.

Looking more closely into the MVPA levels of the caregiving grandparents participating in the present study, significantly lower MPVA levels were found when comparing their care moment with the corresponding non-care moment as specific time frames. However, no within-subject differences in relative MVPA levels were observed between the care day and the non-care day. These results are also in line with the previously mentioned case-control study conducted within the HGP [28], where preliminary findings showed no significant differences between grandparental subgroups for MVPA levels on a weekly basis. Additionally, the results of the present study suggest that grandparents seem to compensate for their lower MVPA levels during the actual care moment since no differences in MVPA levels were found at day level when comparing a day with versus a day without grandchild care provision.

Combining our results with a suggestion made by a previous study, it may thus be plausible that grandparents replace their habitual SB levels with LIPA when providing care for their grandchildren [38]. Additionally, this was partially confirmed in previous research examining determinants influencing grandparents’ PA and SB levels while taking care for their grandchildren [39]. Participating grandparents in this latter qualitative study revealed that they felt the need to be constantly engaged with their grandchild(ren) when in charge of grandchild care, and thus could nearly be sedentary [39]. Additionally, research of Sneed et al. [40] indicated that when grandparents are actively engaged in caregiving tasks, they mostly have to meet the needs of their grandchildren, which may involve various physical tasks (e.g., bending, lifting, sitting and playing on the floor) and/or pursuits (e.g., walking, doing trips, going to a park). Being engaged in these type of activities during grandchild care was also confirmed by the grandparents participating in the previously cited qualitative study [39]. Yet, looking more closely into the intensity of these activities, almost all activities can be categorized as LIPA according to the Compendium of Physical Activities of Ainsworth [41]. Furthermore, it was found that providing care for young(er) children (i.e., aged under 3 years) requires much more focused attention and light intensive care of the caregiver in comparison to providing care for school-aged children [42]. Consequently, since the grandchildren being cared for in the current study had a mean age of only 3.4 years, this could partly clarify the significantly higher grandparental LIPA levels and lower SB levels on the care day when compared to the matching non-care day within the participating grandparents.

Grandparents taking part in the previously mentioned qualitative study also indicated that the limited mobility of young(er) grandchildren (with a restricted range of movement due to not having reached their full potential in terms of locomotion) and the fact that these young(er) grandchildren are not (yet) able to participate in certain activities (such as walking or biking) with the same intensity and distance as the grandparents were used to themselves might lead them to lower levels of MVPA due to the provision of grandchild care [39]. As such, this adaptation to the somewhat less intensive activity levels of the young(er) grandchild(ren) might be a possible explanation for our finding of grandparents’ lower MVPA levels during the actual care moment compared to the non-care moment in the present study. Furthermore, during periods without caregiving responsibilities, grandparents may have more freedom to determine their own activities, with fewer constraints or adjustments related to the grandchild(ren)’s needs, potentially resulting in higher levels of MVPA during those non-care moments.

As the results of the current study demonstrated that providing care for grandchildren in a non-residential setting may act as an unconscious facilitator to improve one’s LIPA levels and decrease one’s SB levels, the provision of grandchild care offers potential benefits in view of other health-related outcomes for grandparents. In this study, grandparents spent an average duration of 342.3 ± 187.0 min with their grandchild(ren) during a care moment. During this average care moment duration and based on the percentages reported, they thus devote an average of 137.0 ± 40.7 min to LIPA compared to 109.9 ± 47.2 min of LIPA during a non-care moment (∆=27.1 min). Additionally, they engaged in SB for an average of 190.7 ± 44.2 min during a care moment as opposed to 213.6 ± 53.4 min during a non-care moment (∆=22.9 min). Evaluating the potential health benefits of the increased levels of LIPA among grandparents is challenging due to the absence of established WHO guidelines for this particular intensity of PA [23]. However, the systematic review by Warburton et al. [43] demonstrated that replacing some SB with LIPA (even for a small number of minutes per day) is effective in obtaining some health benefits. Improving one’s LIPA levels does not require special time commitments or planning, as these activities are typically already part of one’s daily routine [44]. Since grandparents demonstrated higher LIPA levels and lower SB levels on a care day compared to a non-care day, and the fact that this within-subject difference was even more pronounced when comparing the care moment with the non-care moment, grandchild care can potentially contribute to the health of grandparents in a positive way. However, the benefits of caring for grandchildren may extend beyond PA effects in contributing to the health of grandparents. The social interaction inherent to caring for grandchildren facilitates better mental and social well-being, reduced stress and an overall improved quality of life [45, 46]. Moreover, as individuals age, the act of caring for grandchildren becomes even more beneficial as it helps alleviate feelings of loneliness, which is a common issue associated with aging [47, 48]. Furthermore, a substantial 61% of child care facility staff expresses that the workload is excessively demanding [49]. However, alleviating the pressure on child day care centers can be achieved by involving grandparents in the regular care of their grandchildren, offering an additional societal benefit. For example, one could extenuate and encourage the extension of parental leave arrangements to grandparents who are still active on the labor market and providing other (financial) incentives for already retired grandparents, allowing them to assist with child care. Existing policy responses include subsidizing child care provided by grandparents (as in the Netherlands), offering more flexible work schedules or adjusting retirement schemes [38]. Nevertheless, some family experts do not favor the option of too extensive child care provided by grandparents due to their potential negative influence such as isolation or developmental delays for the children themselves as they do not interact with other age-related peers [50]. Additionally, it should be noted that grandparents who provide daily care for their grandchildren may also experience adverse effects on their health due to the increased pressure and stress associated with these educational responsibilities [38]. As such, care moments which are too extensive, long and frequent may leave grandparents with little or no time to compensate for certain behaviors during the remainder of the day, resulting in lower MVPA levels across the day in its entirety which may indirectly affect their overall health [38]. Therefore, future research on the topic should investigate methods to promote grandparents’ (MV)PA levels while providing care for their grandchild(ren). It is also crucial to explore initiatives to ensure that even with intensive caregiving responsibilities, these duties do not adversely affect grandparents’ (MV)PA levels and their related health benefits.

### Strengths and limitations

A first strength of this study is that it is the first one to examine within-subject differences in levels of PA and SB among grandparents at a day and the specific moment within that day when providing care for their grandchild(ren) compared to corresponding times when they do not. Secondly, all data of PA and SB levels were obtained objectively by means of accelerometry, increasing the reliability and accuracy of data collection as well as reducing measurement errors.

A first potential limitation of our study could be seen in the variation with regards to the duration of the (non-)care moments. The types of activities undertaken with the grandchildren during these moments may largely depend on the available time frame and the particular moment of the day during which care was provided. As revealed in an earlier focus group study, grandparents noted that longer care moments tended to facilitate more engagement in PA [39]. For instance, a full day of caregiving seemed to allow grandparents to explore various activities and locations with their grandchildren, resulting in increased PA levels. However, evening care sessions occurring after school, typically involve tasks like bathing the grandchildren, preparing meals and engaging sedentary activities, such as watching movies or reading together [39]. It is imperative to acknowledge that grandparents’ personal experiences may differ in this respect. On the other hand, the fact that – on average – higher levels of LIPA and lower levels of SB were found in grandparents, despite the variable duration of the selected care moments (of at least two consecutive hours), can also be viewed as a strength. Another restriction of this study, however, pertains to the subjective nature of the grandchild care diary reports. The precision and timeliness with which grandparents completed these diaries remains somewhat uncertain. For instance, if grandparents filled out the diary at the end of the measurement week, there is a possibility of recall bias influencing the accuracy of the recorded grandchild care information. However, despite the potential reporting biases, previous research has shown that self-reported measures are valid when used at group level [51]. Moreover, some context factors (e.g., where grandchild care was provided, whether grandparents provided this care alone or with their partner, whether grandparents worked on the selected days or not) were not assessed. Such information could be useful as they may have played a crucial role in the type of activities and the intensity of the activities to engage in with or without the grandchild(ren) [39]. Another aspect to consider is the potential variability in the intensity of school days for school-aged children on weekdays. For instance, Wednesdays in Flanders (Belgium) are typically half days of school, which could influence the range and timing of activities that are feasible to conduct during the provision of grandchild care on that specific day. An illustration of this is that Wednesday afternoons often offer more opportunities for outings and multiple activities, more than likely leading to increased physical engagement for both grandparents and their grandchildren [39]. However, by systematically selecting the first care day of the week meeting all the predefined inclusion criteria, only for one-fourth of the grandparents a Wednesday was selected as the care day to be included in the analyses. Eventually, it is important to consider that the way grandparents care for their grandchild(ren) can differ given diversified family structures and according to sociocultural context within other countries and/or cultures [52, 53]. Therefore, the findings of the current study should be interpreted with caution in terms of their representativeness and generalizability.

## Conclusions

Grandparents exhibited significantly higher levels of LIPA and lower levels of SB during the actual care moment of the care day compared with the corresponding non-care moment on the non-care day. This pattern of within-subject differences was consistent when the care day was compared with the non-care day. The higher percentage of time of LIPA and lower percentage of time spent on SB highlight the positive impact of providing grandchild care among middle-aged and older adults in view of their energy-expenditure related behavior, potentially improving other health-related outcomes in grandparents. Although caring for one’s grandchild(ren) was found to be beneficial for grandparents’ LIPA and SB levels, grandparents also seem to compensate for their lower MVPA levels during the actual caregiving moment during the remainder of the care day. The present study highlights the importance of recognizing caregiving for one’s grandchildren as an important window of opportunity in view of improving middle-aged and older adults’ PA and SB levels as well as the potential benefits thereof in promoting an active lifestyle, contributing to healthy aging.

## Appendix I

**Table 7:** Overview of the number of valid individual data entries over the different assessment time points (*N* = 92), resulting in 166 within-subject comparisons

	*n* (%)
Valid data for T0, T1 & T2	18 (19.6%)
Valid data for T0 only	12 (13.0%)
Valid data for T1 only	14 (15.2%)
Valid data for T2 only	10 (10.9%)
Valid data for T0 & T1, but not T2	14 (15.2%)
Valid data for T1 & T2, but not T3	14 (15.2%)
Valid data for T0 & T2, but not T1	10 (10.9%)

Data were collected at three different assessment time points with a fixed time interval of approximately 6 months in between. N/n: number of individual participants; T0 = baseline, T1 = 6 months; T2 = 12 months

## Appendix II

Within the larger HGP, 162 grandparents participated in the baseline assessment (i.e., T0), 157 in the second assessment time point (i.e., T1) and 162 in the third assessment time point (i.e., T2). Of these participants, respectively 67, 66 and 69 grandparents did not provide care for their grandchild(ren) during the same week in which the accelerometer-derived PA and SB levels were obtained. Additionally, 42 grandparents at T0, 30 grandparents at T1, and 41 grandparents at T2 did not meet the predetermined study inclusion criteria. Table B presents a comprehensive list of the study specific exclusion criteria applied to the grandparental study participants across the three assessment time points.

**Table 8:** Exclusion criteria applied to the grandparental study participants across the three assessment time points

		Time assessment			
Exclusion criteria	T1 (*N* = 42)		T2 (*N* = 30)	T3 (*N* = 41)	
GP only cared for GC older than 5 years during the care day	5		12	11	
The care moment was shorter than 2 consecutive hours	11		3	10	
GP only provided care to their GC during the weekend	9		6	13	
No match for the selected care day with a suitable non-care day was found	4		2	0	
GP provided care to a disparate number of GC during the care moment	13		7	5	
GP provided care for the same number of grandchildren, but at different, non-consecutive periods during the care day	0		0	2	

N: number of excluded participants per assessment time point; GP: grandparent; GC: grandchild(ren)

## Appendix III

**Table 9:** Average duration of PA and SB levels during an average care and non-care day

Outcome variables	Mean ± SD
**Light intensity physical activity (LIPA) (min.day** ^− 1^ **)**	
Care day	306.7 ± 78.7
Non-care day	268.5 ± 85.5
**Moderate-to-vigorous intensity physical activity (MVPA) (min.day** ^− 1^ **)**	
Care day	33.9 ± 30.2
Non-care day	36.4 ± 37.3
**Sedentary behavior (SB) (min.day** ^− 1^ **)**	
Care day	574.0 ± 84.2
Non-care day	605.2 ± 91.0

SD: standard deviation; LIPA: light intensity physical activity; MVPA: moderate-to-vigorous intensity physical activity; SB: sedentary behavior. The total duration of an average care day is 915.6 min and 910.0 min for the average non-care day

**Table 10:** Average duration of PA and SB levels during an average care and non-care moment

Outcome variables	Mean ± SD
**Light intensity physical activity (LIPA) (min.moment** ^− 1^ **)**	
Care moment	137.0 ± 40.7
Non-care moment	109.9 ± 47.2
**Moderate-to-vigorous intensity physical activity (MVPA) (min.moment** ^− 1^ **)**	
Care moment	14.4 ± 23.6
Non-care moment	18.8 ± 27.7
**Sedentary behavior (SB) (min.moment** ^− 1^ **)**	
Care moment	190.7 ± 44.2
Non-care moment	213.6 ± 53.4

SD: standard deviation; LIPA: light intensity physical activity; MVPA: moderate-to-vigorous intensity physical activity; SB: sedentary behavior. The total duration of both the average care moment and corresponding non-care moment is 342.3 min

## Appendix IV

**Fig. 1:**
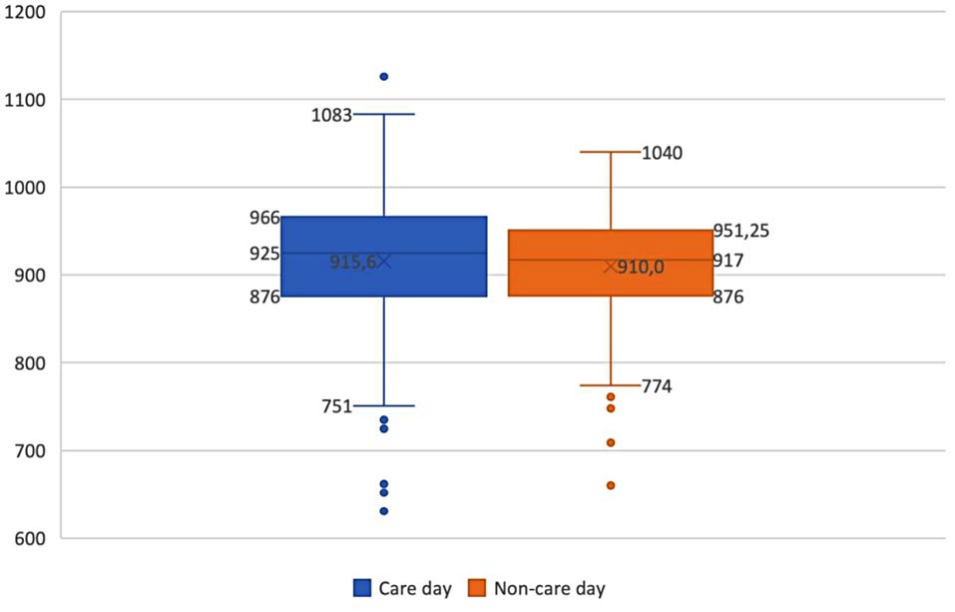
Variability in duration of the care days and non-care days. The horizontal line in the middle of the box represents the median value, while the cross within the box marks the mean. The lower and upper boundaries of the box indicate the 25th and 75th percentiles (i.e., Q1 and Q3), respectively. Whiskers above and below the box indicate the maximum and the minimum values. Outliers, defined as values 1.5 times the interquartile range away from both the upper and lower edges of the box, are indicated by dots

**Fig. 2:**
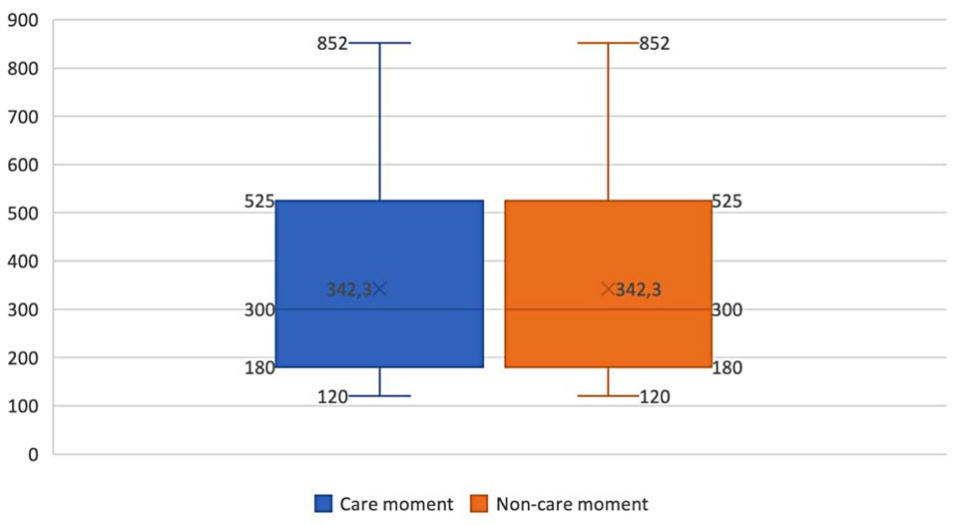
Variability in duration of the care moments and non-care moments. The horizontal line in the middle of the box represents the median value, while the cross within the box marks the mean. The lower and upper boundaries of the box indicate the 25th and 75th percentiles (i.e., Q1 and Q3), respectively. Whiskers above and below the box indicate the maximum and the minimum values

## Data Availability

The datasets used and/or analyzed during the current study are available from the corresponding author upon reasonable request.

## Abbreviations

BMI: Body mass index
GP: Grandparent(s)
HGP: Healthy Grandparenting Project
LIPA: Light intensity physical activity
MPA: Moderate intensity physical activity
MVPA: Moderate-to-vigorous intensity physical activity
PA: Physical activity
SB: Sedentary behavior
SD: Standard deviation
SES: Socio-economic status
VPA: Vigorous intensity physical activity
WHO: World Health Organization

## References

[1] National Research Council (US). Panel on Statistics for an Aging Population. The Aging Population in the Twenty-First Century: Statistics for Health Policy. 1988. 25032446

[2] Wilmoth JR (2000). Demography of longevity: past, present, and future trends. Exp Gerontol.

[3] Flatt T, Partridge L (2018). Horizons in the evolution of aging. BMC Biol.

[4] Oeppen J, Vaupel JW (2002). Demography. Broken limits to life expectancy. Science.

[5] European Commission. The impact of demographic change in europe 2019, June 20 [ https://commission.europa.eu/strategy-and-policy/priorities-2019-2024/new-push-european-democracy/impact-demographic-change-europe_nl#demographic-trends.

[6] Rudnicka E, Napierała P, Podfigurna A, Męczekalski B, Smolarczyk R, Grymowicz M (2020). The World Health Organization (WHO) approach to healthy ageing. Maturitas.

[7] Glaser K, Price D, Di Gessa G, Ribe E, Stuchbury R, Tinker A. Grandparenting in Europe: family policy and grandsparents’ role in providing childcare. Grandparents plus. 2013.

[8] Meulders D, O’Dorchai S. Childcare in Belgium. 2008.

[9] Geurts T, Van Tilburg T, Poortman A-R, Dykstra PA (2015). Child care by grandparents: changes between 1992 and 2006. Ageing Soc.

[10] Hank K, Buber I (2008). Grandparents caring for their Grandchildren: findings from the 2004 survey of Health, Ageing, and Retirement in Europe. J Fam Issues.

[11] Group of Experts on Gender SI. Employment. The provision of childcare services: A comparative review of 30 European countries. 2009.

[12] Bengtson VL (2001). Beyond the Nuclear Family: the increasing importance of multigenerational bonds. J Marriage Family.

[13] Glaser K, Grandparents P, Fundação Calouste G, King’s College L (2010). Beth Johnson F. Grandparenting in Europe.

[14] Szinovacz ME (1998). Grandparents today: a demographic profile. Gerontologist.

[15] Glaser K, Di Gessa G, Tinker A, Grandparents P, King’s College London (2014). Institute of G. Grandparenting in Europe: the health and wellbeing of grandparents caring for grandchildren: the role of cumulative advantage/disadvantage.

[16] Di Gessa G, Glaser K, Tinker A (2016). The impact of caring for grandchildren on the health of grandparents in Europe: a lifecourse approach. Soc Sci Med.

[17] Danielsbacka M, Křenková L, Tanskanen AO (2022). Grandparenting, health, and well-being: a systematic literature review. Eur J Ageing.

[18] Reinkowski J. Should we care that they care? Grandchild Care and its impact on Grandparent Health. ifo Institute - Leibniz Institute for Economic Research at the University of Munich; 2013.

[19] World Health Organisation. Physical activity https://www.who.int/news-room/fact-sheets/detail/physical-activity2022 [.

[20] Peel NM, McClure RJ, Bartlett HP (2005). Behavioral determinants of healthy aging. Am J Prev Med.

[21] Daskalopoulou C, Stubbs B, Kralj C, Koukounari A, Prince M, Prina AM (2017). Physical activity and healthy ageing: a systematic review and meta-analysis of longitudinal cohort studies. Ageing Res Rev.

[22] Seefeldt V, Malina RM, Clark MA (2002). Factors affecting levels of physical activity in adults. Sports Med.

[23] Bull FC, Al-Ansari SS, Biddle S, Borodulin K, Buman MP, Cardon G (2020). World Health Organization 2020 guidelines on physical activity and sedentary behaviour. Br J Sports Med.

[24] Rezende LFMd, Rey-López JP, Matsudo VKR, Luiz OC (2014). Sedentary behavior and health outcomes among older adults: a systematic review. BMC Public Health.

[25] Harvey JA, Chastin SFM, Skelton DA (2013). Prevalence of sedentary behavior in older adults: a systematic review. Int J Environ Res Public Health.

[26] Gennuso KP, Gangnon RE, Matthews CE, Thraen-Borowski KM, Colbert LH. Sedentary behavior, physical activity, and markers of Health in older adults. Med Sci Sports Exerc. 2013;45(8). 10.1249/MSS.0b013e318288a1e5 PMC5764165 23475142

[27] Whitley DM, Fuller-Thomson E, Brennenstuhl S (2015). Health characteristics of Solo Grandparent caregivers and single parents: a comparative Profile using the behavior risk factor Surveillance Survey. Curr Gerontol Geriatr Res.

[28] Vermote M. Healthy grandparenting: The impact of non-residential grandchild care on physical activity and sedentary behavior in people aged 50 years and over. Crazy Copy Center Productions; 2024. 10.1186/s12889-020-10024-9 PMC7789577 33407287

[29] Vermote M, Deliens T, Deforche B, D'Hondt E. The impact of non-residential grandchild care on physical activity and sedentary behavior in people aged 50 years and over: study protocol of the Healthy Grandparenting Project. BMC Public Health. 2021;21(1):38. 10.1186/s12889-020-10024-9 PMC7789577 33407287

[30] Stewart A, Marfell-Jones M, De Olds T. Ridder J. International Standards for Anthropometric Assessment2011.

[31] Braveman PA, Cubbin C, Egerter S, Chideya S, Marchi KS, Metzler M (2005). Socioeconomic status in health research: one size does not fit all. JAMA.

[32] Aadland E, Ylvisåker E (2015). Reliability of the actigraph GT3X + accelerometer in adults under free-living conditions. PLoS ONE.

[33] Freedson PS, Melanson E, Sirard J (1998). Calibration of the Computer Science and Applications, Inc. accelerometer. Med Sci Sports Exerc.

[34] Bates D, Mächler M, Bolker B, Walker S (2015). Fitting Linear mixed-effects models using lme4. J Stat Softw.

[35] Brooks M, Kristensen K, van Benthem K, Magnusson A, Berg C, Nielsen A (2017). glmmTMB balances speed and flexibility among packages for zero-inflated generalized Linear mixed modeling. R J.

[36] Hadley Wickham DN. Thomas Lin Pedersen. ggplot2: Elegant Graphics for Data Analysis (Use R). Ed n. editor: Springer; 2016.

[37] Lüdecke DBA, Schwemmer C, Powell C, Djalovski A, Titz J. sjPlot: Data Visualization for Statistics in Social Science. 2021.

[38] Leimer B, van Ewijk R (2022). Are grandchildren good for you? Well-being and health effects of becoming a grandparent. Soc Sci Med.

[39] Vermote M, Deliens T, Deforche B, D’Hondt E. Determinants of caregiving grandparents’ physical activity and sedentary behavior: a qualitative study using focus group discussions. European Review of Aging and Physical Activity. 2023;20(1):20. 10.1186/s11556-023-00330-7 PMC10601246 37884872

[40] Sneed RS, Schulz R (2019). Grandparent Caregiving, Race, and cognitive functioning in a Population-based sample of older adults. J Aging Health.

[41] Ainsworth BE, Haskell WL, Herrmann SD, Meckes N, Bassett DR, Tudor-Locke C (2011). 2011 Compendium of Physical activities: a second update of codes and MET values. Med Sci Sports Exerc.

[42] Corcoran L, Steinley K, Statistics NCE (2017). Research AIf. Early Childhood Program participation, results from the National Household Education Surveys Program of 2016. First look.

[43] Warburton DER, Bredin SSD (2017). Health benefits of physical activity: a systematic review of current systematic reviews. Curr Opin Cardiol.

[44] Chastin SFM, De Craemer M, De Cocker K, Powell L, Van Cauwenberg J, Dall P (2019). How does light-intensity physical activity associate with adult cardiometabolic health and mortality? Systematic review with meta-analysis of experimental and observational studies. Br J Sports Med.

[45] Kelley SJ, Whitley DM, Escarra SR, Zheng R, Horne EM, Warren GL (2021). The Mental Health well-being of grandparents raising Grandchildren: a systematic review and Meta-analysis. Marriage Family Rev.

[46] Danielsbacka M, Křenková L, Tanskanen AO (2022). Grandparenting, health, and well-being: a systematic literature review. Eur J Ageing.

[47] Yang X, Yin D. The Protective Effect of Caring for Grandchildren on the Mental Health of the Elderly: a structural equation modeling analysis. Int J Environ Res Public Health. 2022;19(3). 10.3390/ijerph19031255 PMC8834749 35162285

[48] Singh A, Misra N (2009). Loneliness, depression and sociability in old age. Ind Psychiatry J.

[49] Statistiek CBvd. Ziekteverzuim en werkdruk in kinderopvang toegenomen na coronaperiode België: Centraal Bureau voor de Statistiek; 2023 [ https://www.cbs.nl/nl-nl/nieuws/2023/06/ziekteverzuim-en-werkdruk-in-kinderopvang-toegenomen-na-coronaperiode#:~:text=In%20de%20kinderopvang%20was%20het,de%20sector%20zorg%20en%20welzijn.

[50] Fergusson E, Maughan B, Golding J (2008). Which children receive grandparental care and what effect does it have?. J Child Psychol Psychiatry Allied Discip.

[51] Portney LG. Foundations of clinical research: applications to evidence-based practice. FA Davis; 2020.

[52] Chan ACY, Lee SK, Zhang J, Banegas J, Marsalis S, Gewirtz AH (2023). Intensity of Grandparent Caregiving, Health, and well-being in Cultural Context: a systematic review. Gerontologist.

[53] Di Gessa G, Glaser K, Price D, Ribe E, Tinker A (2016). What drives National differences in Intensive Grandparental Childcare in Europe?. J Gerontol B Psychol Sci Soc Sci.

